# Association between smoking intensity and duration and tooth loss among Finnish middle-aged adults: The Northern Finland Birth Cohort 1966 Project

**DOI:** 10.1186/s12889-015-2450-6

**Published:** 2015-11-17

**Authors:** Toni Similä, Jorma I. Virtanen

**Affiliations:** Research Unit of Oral Health Sciences, Faculty of Medicine, University of Oulu, P.O. Box 5281, Oulu, FIN-90014 Finland; Medical Research Center Oulu, Oulu, Finland; Oulu University Hospital, Oulu, Finland

**Keywords:** Smoking, Tobacco, Tooth loss, Adult

## Abstract

**Background:**

Smoking is a risk factor for oral diseases and tooth loss. Our aim was to analyze the association between smoking intensity and duration and tooth loss among middle-aged Finnish adults who have enjoyed access to subsidized dental care since childhood.

**Methods:**

This study was based on the Northern Finland Birth Cohort 1966 (NFBC1966) Project, a representative sample of Finnish 46-year-olds. Altogether 1946 46-year-olds participated in a survey and comprehensive clinical oral examinations. We measured smoking exposure in pack-years (intensity) and years of smoking (duration) combined with recent smoking status (current, former, occasional or never). We used negative binomial regression models to estimate the unadjusted and adjusted relative risks (RR) with corresponding 95 % confidence intervals (CI) for tooth loss as an outcome. Gender, education, tooth brushing frequency, dental plaque, diabetes and alcohol use served as explanatory variables for the adjusted models.

**Results:**

Smoking intensity associated with tooth loss in an exposure-dependent manner: those with a high number of pack-years had a significantly greater probability of tooth loss than never smokers: 11–20 pack-years (RR = 1.55, 95 % CI = 1.15–2.08) and 21 or more pack-years (RR = 1.78, 95 % CI = 1.36–2.33). Smoking duration also associated with tooth loss: those who had smoked for several years had a significantly higher probability of tooth loss than never smokers: 21–30 years of smoking (RR = 1.66, 95 % CI = 1.29–2.12) and 31 or more years of smoking (RR = 1.72, 95 % CI = 1.20–2.45).

**Conclusions:**

We found a clear intensity- and duration-dependent relation between smoking and tooth loss among adults with access to subsidized dental care and in good oral health.

## Background

Tobacco smoking is a risk factor for both general and oral diseases [1, 2]. Researchers have identified cigarette smoking as the most important behavioral risk factor for periodontal disease, which in turn is the main cause of tooth loss among the middle-aged and elderly [3–5]. Studies have shown that cigarette smoking associates with fewer remaining teeth and a higher prevalence of edentulousness [6–8].

In addition, researchers have found an exposure-dependent relation between cigarette smoking and tooth loss among young adults [9, 10]. Moreover, a recent study of health professionals revealed a dose-dependent relation, but failed to investigate smoking duration or intensity among current smokers [6]. Among middle-aged Danes who were daily and former smokers, the number of cigarettes smoked associated with tooth loss, and smoking duration associated with tooth loss [11].

When analyzing the effect of smoking, its duration and intensity are the most important aspects to take into account. However, most studies have focused on either the average daily number of cigarettes smoked or years of smoking rather than combining these two measures to represent smoking history. Recently, pack-years have begun to find favor, because this measure takes into account both aspects of smoking with equal weighting (pack-years = years of smoking * the daily consumption of cigarettes/cigarettes per pack (e.g., 20)). Moreover, pack-years or its derivatives have served to reflect the burden of smoking history when the outcome is tooth loss [9, 10]. Few studies have examined the association between different metrics of smoking history (e.g., intensity and duration) and tooth loss with a clinically determined number of missing teeth [4, 11].

In Finland, a National Health Act in the 1970s entitled all young people to free dental care; since then, all Finns have enjoyed access to subsidized dental care, which has greatly improved the oral health of the population. Our aim was to examine the association between smoking and tooth loss among middle-aged Finns with a specific focus on the intensity and duration of smoking history. We used data from the Northern Finland Birth Cohort 1966 Project (NFBC1966), a valuable source of comprehensive information on public and individual health in general [12]. We also examined the association of smoking with tooth loss by gender, education and tooth brushing frequency.

## Methods

### Study design

This cross-sectional study uses data from the longitudinal Northern Finland Birth Cohort 1966 Project (NFBC1966), which comprises a comprehensive sample of babies from the provinces of Lapland and Oulu whose expected birth year was 1966 (12 068 mothers, 12 231 children, 96.3 % of all births in this region) [12]. The Ministry of Social and Health Affairs in Finland approved the data collection, and the Ethics Committee of the Northern Ostrobothnia Hospital District in Oulu, Finland approved the study protocol. We used information from the follow-up study of 46-year-olds (carried out in 2012–2014), which included a postal survey and a comprehensive clinical health examination. In addition, in the Oulu region, the examination also included the full inspection of the mouth and teeth, and during the examination day, participants completed two additional questionnaires. Participation in the follow-up study of 46-year-olds was voluntary, and the participants provided their informed written consent. Altogether 1946 participants (participation rate 62 %) provided information on the number of missing teeth.

### Smoking variables

In 2012, participants received postal questionnaires, which included several questions on previous and current smoking habits, to be returned prior to the clinical health examinations. We calculated pack-years (based on 20 cigarettes per pack) to measure smoking history among those who reported having smoked at least five days a week. In addition, years of smoking served as an alternative measure of smoking history. Both measures of smoking history use equal numbers of participants with available information. We weighted the use of any tobacco product (filtered cigarettes, *n* = 240; other cigarettes, *n* = 22; cigars, *n* = 10; pipe smoking, *n* = 1) equally when calculating pack-years.

Apart from daily smokers, we used separate categories for occasional, former and never smokers. Here, ‘never smokers’ includes all participants who had smoked daily for less than one year in their lifetime and did not smoke at the time of the follow-up. ‘Former smokers’ includes those who had smoked daily for at least one year, but had quit smoking and did not smoke at the time of the study. Those who smoked, but no more than four days a week at the time of the study were considered as ‘occasional smokers’.

### Outcome

Seven dentists who underwent specific training and calibration for this purpose performed the clinical oral examinations. For each participant, the dentists recorded all missing teeth. Excluding third molars, we formed the number of missing teeth variable to measure tooth loss in this study. For the assessment of missing teeth, the kappa values for inter- and intra-examiner agreements were 1.00 and 0.97, respectively. In addition, we defined a dichotomous variable: ‘missing one or more teeth’ or ‘none’ (53 % were missing at least one tooth).

### Explanatory variables

The demographic variables included gender and education. Postal questionnaires inquired about education with two questions: one for comprehensive school and the matriculation exam, and the other for vocational training. Based on these questions, we defined a three-class ordinal variable. ‘Basic education’ included those who had not graduated from high school and had no formal vocational qualifications. ‘Secondary education’ included those who had graduated from high school or vocational school. ‘Higher education’ comprised participants with a university degree or those who had graduated from a polytechnic or equivalent school.

From the postal questionnaires we also determined tooth brushing frequency and use of alcohol. For tooth brushing frequency, we dichotomized the information on the original variable (with five categories) according to the general recommendation of brushing twice daily: ‘once daily or less’ or ‘at least twice daily’ [13]. To avoid missing data, we used similar questions in an additional questionnaire (completed on the day of the health examination), in case corresponding information was missing in the postal questionnaire, which served as the primary source for all the information. Alcohol use was inquired with several questions on the number of consumed standard doses and events of different beverages separately (mild alcoholic beverages: beer, cider and long drink; wine and spirits). We used the classification for alcohol contents per standard doses of different beverages by Sundell et al. [14] and calculated a continuous grams per week variable. Alcohol drinkers were defined as those consuming >230 g/wk for men and >150 g/wk for women, and the rest were defined as light drinkers.

We determined diabetes status using numerous sources: self-reported physician-diagnosed diabetes and medications from the postal questionnaires, hospital outpatient and inpatient registers, and medication registers from the Social Insurance Institution of Finland. The definition of the dichotomous variable (yes/no) did not distinguish between types 1 and 2 diabetes.

Dental plaque served as an indicator of oral health. During the oral health examination, the dentists recorded plaque status (‘none’, ‘visible plaque or plaque detected while probing’) for all visible teeth (excluding third molars) and then, for simplicity, we dichotomized this information (yes/no).

### Statistical analysis

We used a negative binomial regression model for the number of missing teeth as a count variable and then calculated unadjusted and adjusted relative risks (RR) with 95 % confidence intervals (CI) for each explanatory variable in the model in question [15]. In addition, we checked over all two-way interaction terms for explanatory variables.

For the count outcome, we performed stratified analyses by gender, education and tooth brushing frequency. Here, we narrowed education as a stratification variable to low and high so that the low stratum included basic and secondary education, and the high stratum included those with a higher education level in our original education variable.

We performed additional analyses to illustrate the correlation between continuous pack-years and the number of missing teeth as the count variable among those who had smoked. For visual preference, this illustration was based on an unadjusted negative binomial model.

We used the statistical package R environment version 3.1.2 for all statistical analyses [16]. For negative binomial modeling, we used the glm.nb function (with no offset option) in the MASS package.

## Results

Table 1 shows the basic characteristics of the study population and the mean number of missing teeth as well as the proportion of those who were missing at least one tooth according to the categories of study variables and smoking status. The mean number of missing teeth in the entire study population varied with education level (ranging from 1.1 at the higher level to 2.2 at the basic level), tooth brushing frequency (1.7 in those who brushed their teeth once daily or less and 1.2 in those who brushed their teeth at least twice daily), and diabetes status (1.9 in those who had diabetes and 1.4 in those who did not have diabetes). The proportions of those with a basic or secondary education, a habit of alcohol use and poor tooth brushing frequency were higher among current and former smokers than among never smokers. Table 2 shows the distribution of pack-years and years of smoking as well as the association with the number of missing teeth in a similar manner to Table 1.

**Table 1:** Basic characteristics of the study population and number of missing teeth per participant by smoking

	Current smoker^a^			Former smoker^b^			Never smoker^c^			All		
	(*n* = 424)			(*n* = 448)			(*n* = 877)			(*n* = 1946)		
	Participants	Number of missing teeth		Participants	Number of missing teeth		Participants	Number of missing teeth		Participants	Number of missing teeth	
	%	Mean	≥1: %	%	Mean	≥ 1: %	%	Mean	≥ 1: %	%	Mean	≥ 1: %
Gender (*n* = 1946)												
Male	50	2.1	63	54	1.6	57	41	1.1	45	47	1.5	53
Female	50	1.6	62	46	1.2	53	59	1.2	50	53	1.3	53
Education (*n* = 1865)												
Basic	8	2.8	82	6	1.8	62	3	1.8	65	5	2.2	70
Secondary	48	2.1	67	42	1.8	63	28	1.5	59	36	1.7	63
Higher	44	1.3	55	52	1.1	48	69	1.0	42	59	1.1	46
Tooth brushing (*n* = 1944)												
Once daily or less	40	2.0	65	39	1.7	61	26	1.4	52	34	1.7	58
At least twice daily	60	1.7	61	61	1.2	51	74	1.0	46	66	1.2	51
Plaque (*n* = 1935)												
Yes	81	1.9	63	80	1.4	55	78	1.1	47	79	1.4	54
No	19	1.7	59	20	1.5	53	22	1.1	49	21	1.3	52
Diabetes (*n* = 1919)												
Yes	4	1.7	65	2	1.7	43	2	2.3	56	2	1.9	58
No	96	1.8	62	98	1.4	55	98	1.1	48	98	1.4	53
Alcohol use, g/wk (*n* = 1891)												
Alcohol drinker^e^	19	2.2	68	13	1.4	47	6	1.3	48	10	1.7	57
Light drinker^d^	81	1.7	61	87	1.4	56	94	1.1	48	90	1.3	53
Total (*n* = 1946)	100	1.8	62	100	1.4	55	100	1.1	48	100	1.4	53

^a^Those who smoked at the time of the survey

^b^Those who have smoked at least for a year but did not smoke at the time of the survey

^c^Those who had never smoked, or have smoked less than a year and did not smoke at the time of the survey

^d^0–230 g/wk for men and 0–150 g/wk for women

^e^ > 230 g/wk for men and >150 g/wk for women

**Table 2:** Observed distribution of the participants and information on the distribution of outcome by smoking

	Participants	Number of missing teeth	
	%	Mean	≥ 1: %
0–10 pack-years	4	1.6	60
11–20 pack-years	5	2.1	69
21 or more pack-years	6	2.4	67
Former smoker	26	1.4	55
Occasional smoker	8	1.2	54
Never smoker	51	1.1	48
1–20 years of smoking	4	1.8	62
21–30 years of smoking	8	2.2	68
31 or more years of smoking	3	2.4	66
Total (*n* = 1735)	100	1.4	53

Table 3 shows the unadjusted and adjusted relative risks (RR) for the average increase in the number of missing teeth for the entire study population. Smoking 21 or more pack-years associated with tooth loss (adjusted RR = 1.78, CI = 1.36–2.33). Similarly, 31 or more years of smoking associated with the outcome (adjusted RR = 1.72, CI = 1.20–2.45). From other study variables, education in particular associated with the outcome: adjusted RRs for basic and secondary education were 1.81 (CI = 1.34–2.46) and 1.48 (CI = 1.27–1.72), respectively. In addition, poor tooth brushing frequency associated slightly with the outcome (adjusted RR = 1.19, CI = 1.02–1.39).

**Table 3:** Results of the negative binomial regression analyses for the risk of losing teeth (*n* = 1694). Unadjusted and adjusted^a^ relative risks (RR) with 95 % confidence intervals (CI)

	Unadjusted RR (95 % CI)	Adjusted RR (95 % CI)
Gender		
Male	1.16 (1.01–1.34)	0.96 (0.83–1.11)
Female (reference)	1.00	1.00
Education		
Basic	2.11 (1.56–2.84)	1.81 (1.34–2.46)
Secondary	1.67 (1.45–1.93)	1.48 (1.27–1.72)
Higher (reference)	1.00	1.00
Tooth brushing		
Once daily or less	1.38 (1.19–1.59)	1.19 (1.02–1.39)
At least twice daily (reference)	1.00	1.00
Plaque		
Yes	1.06 (0.89–1.26)	0.97 (0.82–1.15)
No (reference)	1.00	1.00
Diabetes		
Yes	1.50 (0.97–2.33)	1.40 (0.92–2.15)
No (reference)	1.00	1.00
Smoking		
0–10 pack-years	1.50 (1.06–2.13)	1.39 (0.99–1.97)
11–20 pack-years	1.85 (1.38–2.49)	1.55 (1.15–2.08)
21 or more pack-years	2.18 (1.67–2.85)	1.78 (1.36–2.33)
Former smoker	1.25 (1.06–1.48)	1.13 (0.96–1.34)
Occasional smoker	1.08 (0.83–1.41)	0.98 (0.75–1.28)
Never smoker (reference)	1.00	1.00
1–20 years of smoking	1.62 (1.15–2.28)	1.40 (0.99–1.96)
21–30 years of smoking	2.00 (1.57–2.55)	1.66 (1.29–2.12)
31 or more years of smoking	2.00 (1.40–2.86)	1.72 (1.20–2.45)
Never smoker (reference)	1.00	1.00
Alcohol use, g/wk		
Alcohol drinker	1.25 (1.01–1.55)	1.09 (0.88–1.36)
Light drinker (reference)	1.00	1.00

^a^Adjusted for other background factors in this table. In addition, other than smoking variables were adjusted for smoking (pack-years)

Table 4 shows the relation of smoking to tooth loss stratified according to gender, education and tooth brushing frequency. The RRs revealed an exposure-dependent association between pack-years and years of smoking and tooth loss among all the strata, except for women. Adjusted RRs, however, revealed similar, though statistically non-significant, associations among women. In each stratum, former or occasional smokers showed no essential differences from never smokers.

**Table 4:** Results of the stratified negative binomial regression analyses for the risk of losing teeth (n = 1694). Adjusted^a^ relative risks (RR) with 95 % confidence intervals (CI)

	Gender		Education		Tooth brushing	
	Male	Female	Low	High	Once daily or less	At least twice daily
	(*n* = 785)	(*n* = 909)	(*n* = 688)	(*n* = 1006)	(*n* = 557)	(*n* = 1137)
	RR (95 % CI)	RR (95 % CI)	RR (95 % CI)	RR (95 % CI)	RR (95 % CI)	RR (95 % CI)
Smoking						
0–10 pack-years	1.90 (1.12–3.21)	1.07 (0.68–1.70)	1.34 (0.84–2.14)	1.42 (0.85–2.37)	1.58 (0.85–2.95)	1.35 (0.89–2.04)
11–20 pack-years	2.27 (1.41–3.65)	1.20 (0.83–1.74)	1.65 (1.16–2.35)	1.35 (0.79–2.30)	1.97 (1.21–3.23)	1.31 (0.90–1.91)
21 or more pack-years	2.23 (1.52–3.26)	1.41 (0.96–2.08)	1.64 (1.20–2.25)	2.25 (1.35–3.74)	1.81 (1.21–2.70)	1.81 (1.25–2.63)
Former smoker	1.36 (1.06–1.75)	0.98 (0.78–1.24)	1.14 (0.90–1.44)	1.12 (0.88–1.43)	1.22 (0.92–1.61)	1.09 (0.88–1.34)
Occasional smoker	1.24 (0.86–1.80)	0.76 (0.52–1.12)	0.99 (0.67–1.45)	1.01 (0.70–1.46)	0.95 (0.62–1.44)	1.01 (0.71–1.42)
Never smoker (reference)	1.00	1.00	1.00	1.00	1.00	1.00
1–20 years of smoking	2.00 (1.17–3.42)	1.07 (0.69–1.65)	1.47 (0.96–2.24)	1.28 (0.73–2.25)	1.60 (0.89–2.88)	1.32 (0.87–2.00)
21–30 years of smoking	2.15 (1.49–3.11)	1.32 (0.95–1.84)	1.68 (1.25–2.26)	1.56 (1.00–2.44)	1.85 (1.25–2.72)	1.55 (1.12–2.14)
31 or more years of smoking	2.33 (1.40–3.87)	1.26 (0.76–2.08)	1.47 (0.95–2.29)	2.35 (1.31–4.22)	1.95 (1.13–3.36)	1.59 (0.98–2.56)
Never smoker (reference)	1.00	1.00	1.00	1.00	1.00	1.00

^a^Adjusted for diabetes, dental plaque, alcohol use and other stratification factors (gender, education, tooth brushing) in this table

**Fig. 1:**
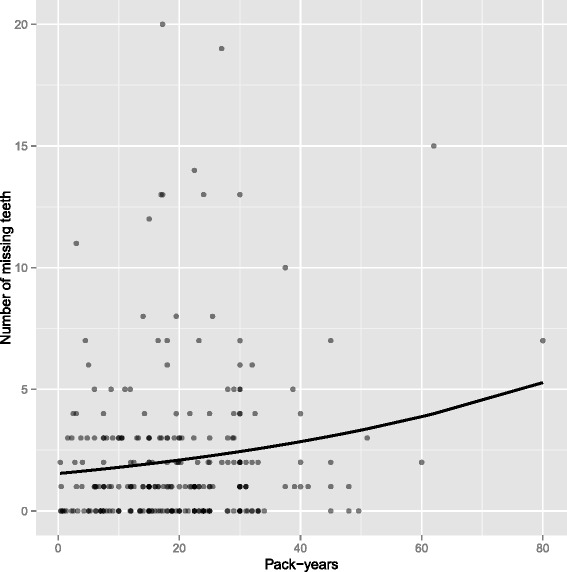
Expected (line) and observed (dots) number of missing teeth by pack-years among smokers. The estimation is based on an unadjusted negative binomial model

The model-based estimate of the number of missing teeth among smokers according to pack-years appears in Fig. 1. This visualization shows the possibility of an exponentially increasing probability to lose teeth with increasing pack-year values.

## Discussion

We used both pack-years and years of smoking to explore the relation between smoking history and tooth loss, and found a clear exposure-dependent association with tooth loss among middle-aged Finnish adults. Among those middle-aged Finnish adults with fairly good oral health, the risk for tooth loss increased significantly from 11 pack-years and with a history of 21 or more years of smoking, especially among males irrespective of their socio-economic background. Those who had stopped smoking or smoked only occasionally were at no higher risk for tooth loss than never smokers, thus substantiating the benefit of smoking cessation.

### Comparison with other studies

Despite differences in studies of smoking and tooth loss in various populations, previous studies have pointed to an exposure-dependent relation between tobacco smoking history and tooth loss. Our findings are in line with those of previous research on smoking and tooth loss [4, 6, 9, 10]. Ojima et al. [10] found an exposure-related association between smoking and tooth loss among young Japanese adults. Previously, with the investigation of the association between smoking and tooth loss in the 31-year-old NFBC1966 cohort, smoking showed an exposure-dependent relation with tooth loss [9]. However, that study involved no oral health examinations, so self-reported postal questionnaires provided data on the number of teeth.

Most previous studies on smoking and tooth loss have not assessed the intensity and duration of smoking in the same study and have used a binary variable rather than the original scale of the number of teeth for tooth loss as an outcome [9, 10]. Our study takes advantage of both of these measures of smoking, which strengthens our findings over those of previous studies. Moreover, we used a count outcome for tooth loss and relative risk (RR), which are considered more accurate measures than the more commonly used odds ratio (with binary outcome) to assess association in cross-sectional studies. Mai et al. [4] used multiple measures of smoking history to investigate its association with tooth loss, but the study was limited to postmenopausal women, and ‘any tooth loss’ served as a binary outcome. Although the study on middle-aged Danes shares similarities with our study, Morse et al. [11] did not use pack-years (or any other corresponding measure) to assess smoking intensity and they used only a binary outcome for tooth loss (6+ versus <6 teeth lost).

Education and tooth brushing frequency also associated significantly with tooth loss in our study. In particular, the impact of socio-economic status (SES), measured as education level, on tooth loss seemed closely resembled that of smoking. Previous studies have also revealed a strong association between low education and tooth loss [4, 8, 17]. In addition, a low level of oral self-care, commonly assessed as a tooth brushing frequency of once daily or less, has shown a stronger connection with a lower number of present teeth than has a high level of oral self-care [9, 18]. However, this connection has proved to be relatively weak, and some studies have found no significant association between tooth brushing frequency and tooth loss among either gender [10, 19].

Alcohol use is known to accompany smoking habit often and it may act as a confounder for the association between smoking and oral diseases [20]. However, in our study alcohol use was only weakly associated with the outcome. Moreover, we performed interaction analyses with alcohol use and smoking (pack-years and current smoking habit) and did not observe statistically significant interaction. Previous studies have shown inconsistent results for how alcohol use associates with tooth loss [11, 21].

The rate of tooth loss in our study was similar among both men and women, a finding in line with those of previous studies [9–11, 22]. However, this association depends strongly on other factors, such as age and population, which have led to a wide range of observations across studies [8, 18, 23]. Since all the NFBC1966 members were the same age, we were unable to investigate the influence of age beyond reporting the prevalence of tooth loss at this age. Nevertheless, aging is known associate strongly with tooth loss.

In our study, the cohort members were among the first people to receive comprehensive Finnish public health care, including free oral care from childhood, as a result of the National Health Act of 1972 [24]. Those born in the late 1960s were the first to benefit from this new, free-of-charge public oral health care from the beginning of school (i.e., the age when the first permanent teeth erupt) through adolescence. Since then, the cohort participants have enjoyed heavily subsidized dental care throughout their lifetime. Generally, the oral health of these 46-year-old adults was better than that of the roughly same-age participants from the previous Health 2000 Survey in Finland [25]. In our study, 53 % of the participants had experienced tooth loss (not counting third molars).

### Strengths and limitations

The comprehensive and representative data on the 46-year-old Finnish adult population is one strength of this study. The NFBC1966 cohort study has been a unique research project, collecting detailed information on cohort members across several life stages. We had information (about tooth loss) from 1946 participants, with fairly even representation of men (47 %) and women (53 %). Smoking variables were sufficiently thorough to assess the participants’ smoking history in detail (smoking duration, amount, and intensity were calculable). Moreover, for the first time, the follow-up of 46-year-olds included complete clinical oral examinations, which enabled the clinical measurement of the number of missing teeth rather than self-reported measurements. Self-reported tooth loss has been considered an acceptable substitute for clinically measured tooth loss, but some validity issues remain for participants with certain characteristics [26].

Due to the cohort study design, with its several follow-ups and health monitoring, the prevalence of many oral health-related diseases and symptoms, as well as the presence of associated lifestyle-related risk factors, may be lower among cohort members than in the general Finnish population [25]. Another weakness relates to the follow-up data on the 46-year-olds: we were able to examine only the association between smoking and tooth loss at this age and thus cannot predict how strong the association might have been in a younger or older study population.

In this study, the cause of tooth loss was unavailable. In addition, we do not know the exact moment of tooth loss, which raises the possibility that some of the individuals’ outcomes may have preceded their exposure. However, this scenario among these middle-aged adults with fairly good oral health is unlikely. Although we adjusted for common confounding factors, some problems related to the accuracy of the results may persist, possibly due to unknown risk factors or errors in the self-reported or other measurements. For instance, because we used only education as a measure for socio-economic status and tooth brushing for oral health behavior [9, 18], one should exercise caution when interpreting the findings.

## Conclusions

We demonstrated the impact of smoking history and current smoking status on tooth loss in a population of 46-year-old adults and found that the probability of losing teeth increases in an exposure-dependent manner commensurate with smoking intensity and duration. Conversely, those who had stopped smoking or smoked only occasionally were at lower risk for losing teeth than those who smoked daily. Thus, smoking cessation and quitting smoking among adults should be a priority.

## Abbreviations

CI: Confidence interval
NFBC1966: the Northern Finland Birth Cohort 1966
RR: Relative risk
SES: Socio-economic status

## References

[1] Reibel J (2003). Tobacco and oral diseases. Update on the evidence, with recommendations. Med Princ Pract.

[2] Agnihotri R, Gaur S (2014). Implications of tobacco smoking on the oral health of older adults. Geriatr Gerontol Int.

[3] Palmer RM, Wilson RF, Hasan AS, Scott DA (2005). Mechanisms of action of environmental factors—tobacco smoking. J Clin Periodontol.

[4] Mai X, Wactawski-Wende J, Hovey KM, LaMonte MJ, Chen C, Tezal M, Genco RJ (2013). Associations between smoking and tooth loss according to the reason for tooth loss: the Buffalo OsteoPerio Study. J Am Dent Assoc.

[5] Püllen F, Folberth R, Ruhmann C, Eickholz P (2013). Tooth extractions in general and due to periodontal reasons in three dental practices: a case-control study. Quintessence Int.

[6] Dietrich T, Maserejian NN, Joshipura KJ, Krall EA, Garcia RI (2007). Tobacco use and incidence of tooth loss among US male health professionals. J Dent Res.

[7] Krall EA, Dawson-Hughes B, Garvey AJ, Garcia RI (1997). Smoking, smoking cessation, and tooth loss. J Dent Res.

[8] Haikola B, Oikarinen K, Söderholm AL, Remes-Lyly T, Sipilä K (2008). Prevalence of edentulousness and related factors among elderly Finns. J Oral Rehabil.

[9] Ylöstalo P, Sakki T, Laitinen J, Järvelin MR, Knuuttila M (2004). The relation of tobacco smoking to tooth loss among young adults. Eur J Oral Sci.

[10] Ojima M, Hanioka T, Tanaka K, Aoyama H (2007). Cigarette smoking and tooth loss experience among young adults: a national record linkage study. BMC Public Health.

[11] Morse DE, Avlund K, Christensen LB, Fiehn NE, Molbo D, Holmstrup P, Kongstad J, Mortensen EL, Holm-Pedersen P (2014). Smoking and drinking as risk indicators for tooth loss in middle-aged Danes. J Aging Health.

[12] Conference on Epidemiological Birth Cohort Studies. Paula Rantakallio Memorial Symposium, 2014. http://www.oulu.fi/sites/default/files/Rantakallio_conference_2014_abstract_book.pdf. Accessed 13 Nov 2015.

[13] Delivering better oral health: an evidence-based toolkit for prevention. Third Edition. Public Health England 2014. https://www.gov.uk/government/uploads/system/uploads/attachment_data/file/367563/DBOHv32014OCTMainDocument_3.pdf. Accessed 13 Nov 2015.

[14] Sundell L, Salomaa V, Vartiainen E, Poikolainen K, Laatikainen T (2008). Increased stroke risk is related to a binge drinking habit. Stroke.

[15] Zeileis A, Kleiber C, Jackman S (2008). Regression models for count data in R. J Stat Soft.

[16] The R Project for Statistical Computing. http://www.r-project.org. Accessed 13 Nov 2015.

[17] Mundt T, Polzer I, Samietz S, Grabe HJ, Dören M, Schwarz S, Kocher T, Biffar R, Schwahn C (2011). Gender-dependent associations between socioeconomic status and tooth loss in working age people in the Study of Health in Pomerania (SHIP), Germany. Community Dent Oral Epidemiol.

[18] Kim HN, Ha TG, Kim MJ, Jun EJ, Jeong SH, Kim JB. Factors related to number of present teeth in Korean elderly adults aged 55-84 years. Int J Dent Hyg. 2015; doi:10.1111/idh.12151. 10.1111/idh.12151 26074207

[19] Morita I, Nakagaki H, Toyama A, Hayashi M, Shimozato M, Watanabe T, Tohmatsu S, Igo J, Sheiham A (2006). Behavioral factors to include in guidelines for lifelong oral healthiness: an observational study in Japanese adults. BMC Oral Health.

[20] Ferreira Antunes JL, Toporcov TN, Biazevic MG, Boing AF, Scully C, Petti S (2013). Joint and independent effects of alcohol drinking and tobacco smoking on oral cancer: a large case-control study. PLoS One.

[21] Åstrøm AN, Gülcan F, Ekbäck G, Ordell S. Long-term healthy lifestyle patterns and tooth loss studied in a Swedish cohort of middle-aged and older people. Int J Dent Hyg. 2015;13:292–300. 10.1111/idh.12173 26294114

[22] Ito K, Aida J, Yamamoto T, Ohtsuka R, Nakade M, Suzuki K, Kondo K, Osaka K, JAGES Group (2015). Individual- and community-level social gradients of edentulousness. BMC Oral Health.

[23] Desvarieux M, Schwahn C, Völzke H, Demmer RT, Lüdemann J, Kessler C, Jacobs DR, John U, Kocher T (2004). Gender differences in the relationship between periodontal disease, tooth loss, and atherosclerosis. Stroke.

[24] Järvelin J. Health care systems in transition: Finland. European Observatory on Health Care Systems, 4(1). Copenhagen: WHO Regional Office for Europe; 2002. http://www.euro.who.int/document/e74071.pdf. Accessed 13 Nov 2015.

[25] Suominen-Taipale L, Nordblad A, Vehkalahti M, Aromaa A (2008). Oral health in the Finnish adult population. Health 2000 Survey B25/2008.

[26] Gilbert GH, Chavers LS, Shelton BJ (2002). Comparison of two methods of estimating 48-month tooth loss incidence. J Public Health Dent.

